# Ethanol marshall bundle elimination, pulmonary vein isolation, and linear ablation for atrial fibrillation with or without heart failure

**DOI:** 10.3389/fcvm.2024.1486621

**Published:** 2024-11-25

**Authors:** Hongxu Chen, Huahua Li, Dan Chen, Xiong Xiong, Xi Li, Yanhong Chen, Jinlin Zhang

**Affiliations:** ^1^ Department of Cardiology, Wuhan Asia Heart Hospital Affiliated to Wuhan University of Science and Technology, Wuhan, Hubei, China; ^2^ Department of Medicine, School of Medicine, Wuhan University of Science and Technology, Wuhan, Hubei, China; ^3^ Department of Cardiology, Wuhan Asia General Hospital, Wuhan, Hubei, China; ^4^ Division of Cardiac Arrhythmia, Cardiac and Vascular Center, The University of Hong Kong-Shenzhen Hospital, Shenzhen, China

**Keywords:** atrial fibrillation, heart failure, catheter ablation, vein of marshall, esophageal fistula

## Abstract

**Background:**

In medical practice, atrial fibrillation (AF) is intricately associated with heart failure (HF). Currently, ethanol infusion of vein of Marshall (EIVOM) for AF ablation in HF patients remains significantly limited.

**Method:**

This was a non-randomized, single-center, retrospective observational study. AF patients received 4-step ablation composed of EIVOM, pulmonary vein isolation (PVI) and linear ablation. The primary composite endpoint was defined as recurrence of atrial tachycardia over 30 s. Propensity score matching (PSM) was performed to reduce selection bias.

**Results:**

From April 2020 to May 2022, 362 patients were included, comprising of 182 HF patients and 180 non-HF patients. EIVOM success rate was lower in HF patients than non-HF patients (86.8% vs. 93.9%). Cardiac effusion was more common in HF patients (44.0% vs. 37.2%), and 2 cases of atrial-esophageal fistula were observed in the HF group. During a median follow-up of 12 months, no significant difference in the primary endpoint was observed between HF and non-HF group. Different HF subgroups had similar AF recurrence. After PSM, AF recurrence rate remained statistically equivalent between the HF and non-HF groups.

**Conclusion:**

EVIOM combining catheter ablation can be completed with comparable success rate in AF patients with or without HF. However, peri-procedural safety is a concern for HF patients undergoing EIVOM combing AF catheter ablation. During the follow-up, HF status before ablation is not related with increased AF recurrence.

## Introduction

Atrial fibrillation (AF) is the most common arrhythmia encountered in clinical practice. AF and heart failure (HF) share common risk factors and are interrelated as both cause and effect, often coexisting (1). Patients with both AF and HF face worse cardiac function and lower quality of life (1). HF is also the leading cause of death within one year for patients who have sought emergency care due to AF (2).

Catheter ablation is effective in lowering AF burden in HF patients and yields a better prognosis. The CASTLE-AF study showed that for patients with AF and left ventricular ejection fraction (LVEF) less than 35%, catheter ablation significantly reduced the composite endpoint of all-cause mortality and hospitalization for worsening HF (3). The AATAC study reported that for HF patients with an LVEF less than 40%, catheter ablation reduced mortality and rehospitalization rates (4). For patients with end-stage HF and AF, the CASTLE-HTx study also demonstrated the advantages of catheter ablation over medical therapy for the composite endpoints of death, left ventricular assist device implantation, and heart transplantation (5, 6). For AF patients with preserved ejection fraction HF, catheter ablation can improve hemodynamic parameters, exercise tolerance, and quality of life (7, 8).

Nevertheless, there are concerns that pulmonary vein isolation (PVI) alone yields low success rate in HF patients (9). Various additional ablation strategies are currently being explored.

Ethanol infusion of the vein of Marshall (EIVOM) is a novel technique that has been proposed as an adjunctive therapy for AF ablation. Marshall bundle (MB) elimination combined with PVI and linear ablation is a novel ablation strategy that systematically targets anatomical atrial structures (VOM ethanol infusion, PVI, and prespecified linear lesions) which are involved in the initiation and maintenance of AF. this comprehensive approach aims to disrupt abnormal electrical pathways and restore normal sinus rhythm. Researches show that it increased success rate in patients with persistent AF, compared to traditional ablation strategy (10–13). However, the efficacy and safety of this strategy remains unexplored in AF patients complicated with HF.

In this article, we aim to report our initial experience with MB elimination + PVI + linear ablation in AF patients with or without HF.

## Method

### Study design

This is a single-center, retrospective analysis conducted at Wuhan Asia Heart Hospital. Patients who underwent catheter ablation of AF combined with EIVOM were consecutively enrolled. Exclusion criteria included left atrial or left atrial appendage thrombosis, cancer patients with a life expectancy less than 1 year, and AF caused by reversible factors.

The study protocol was reviewed and approved by the Institutional Review Board of Wuhan Asia Heart Hospital. The study complied with the Declaration of Helsinki and was approved by the medical ethical committee of Wuhan Asia Heart Hospital, China (No: 2018-YXKY-B017). Data were collected retrospectively and analyzed anonymously. As this was a retrospective analysis, patients' informed consent was waived by the institutional review board (The medical ethical committee of Wuhan Asia Heart Hospital, China).

### Baseline clinical characteristics

Baseline clinical characteristics were retrospectively collected, including age, gender, body mass index (BMI), history of hypertension, diabetes mellitus, coronary artery disease, history of cardiomyopathy, history of ischemic stroke/transient ischemic attack (TIA), New York heart association (NYHA) heart function class, glomerular filtration rate (GFR), serum creatinine, N-terminal B-type natriuretic peptide (NT-ProBNP), CHA2DS2-VASc score, HAS-BLED score, echocardiography, and medications.

### Diagnosis of HF

HF was diagnosed according to the following criteria. 1. Patients had clinical signs or symptoms of HF, 2. Patient's NT-proBNP was ≥600 pg/ml, 3. Patients had at least 1 echocardiographic sign suggestive of HF (EF <50%, enlarged left ventricular volume due to LV systolic dysfunction, systolic pulmonary artery pressure via tricuspid regurgitation, severe left atrial dysfunction due to LV diastolic dysfunction, etc). HF was classified into HFpEF and HFrEF according to their left ventricular ejection fraction (LVEF) measured with TTE. HFrEF was diagnosed when LVEF was less than 50%. And the diagnosis of HFpEF was based on the patient's preserved LVEF (>50%).

### Ablation strategy

For each patient, the procedure was performed under general anesthesia. All patients followed a 4-step ablation strategy, including ethanol Marshall Bundle elimination, pulmonary vein isolation, and Anatomical Linear Ablation. The ablation procedure was described as below.

Step 1: EIVOM

Coronary sinus (CS) angiography was conducted prior to AF catheter ablation to accurately identify the vein of Marshall (VOM). In cases where the VOM was present, an angioplasty wire was carefully inserted into this vein and then a guiding catheter was used to successfully navigate and cannulate the VOM. An over-the-wire (OTW) balloon was introduced into the VOM. 8–10 ml of ethanol was infused into the VOM through the OTW balloon. Effect of EIVOM was assessed with voltage mapping of left atria (LA) afterward.

Step 2: Wide antral PVI

AF catheter ablation was guided with a 3D mapping system: CARTO-3 system (Biosense Webster, Diamond Bar, CA, USA) or the Rhythmia mapping system (Boston Scientific, MA). Point-by-point ablation was performed in a power-control mode (temperature 43°C; saline irrigation 15 ml/min). For CARTO-3 system, automated lesion (AI) annotation was performed using the VisiTag module with AI targets set at 450 for LA roof/floor (45 W, temperature 43°C), 450–500 for LA ridge (45 W, temperature 43°C) and 380 for LA posterior wall (45 W, temperature 43°C). Endpoint of ablation was defined as complete elimination of pulmonary vein (PV) muscle-sleeve potential, including potentials from the PV carina.

Step 3: LA roof linear ablation

LA roof linear ablation was performed through the LA roof linking the two isolated PVI ring. The target AI was set at 400 and ablation energy was 35 W. Blockage of the line was checked and additional ablation was performed to close the gap on the roof line.

Step 4: Mitral isthmus (MI) linear ablation

Linear ablation was performed across the MI from mitral annulus to the left inferior PV with the ablation energy of 35 W. The target AI was set at 450. Epicardial ablation within the CS (25 W) was performed if the MI line blockage was not achieved with endocardial ablation.

Tricuspid isthmus ablation, anterior LA linear ablation, or complex fractionated atrial electrograms (CFAEs) were not routinely performed. The decision to perform these ablations was based on the mechanism of the tachycardias, as well as the electro-substrate of the left atrium.

### Post-procedural management

After the operation, patients were transferred to the cardiac care unit (CCU) for anesthesia recovery. They also received a TTE at CCU to rule out cardiac effusion. Patients were monitored for 4–5 days before discharge. Anticoagulation therapy with warfarin or novel oral anticoagulants (NOAC) was used for at least 3 months after the procedure, and patients taking warfarin maintained an international normalized ratio (INR) between 2 and 3. After 3 months, all patients underwent a reassessment to evaluate their risk of stroke. This assessment was crucial in determining whether it was necessary to continue their anticoagulation therapy. Anticoagulant therapy was continued after the blanking period if the patient's CHA2DS2-VASc score was 2 or higher. Antiarrhythmic drugs, such as amiodarone and beta blockers, were routinely given within 3 months after the procedure to reduce the recurrence of arrhythmia. Proton pump inhibitors were given for 1 month after the procedure. In consideration of the patient's risk factors and prevalent medical conditions, additional therapeutic agents were chosen after the blanking period (3 months after the index ablation).

### Follow-up

Follow-up were completed at outpatient clinic 3, 6, and 12 months after the procedures. Medical history was obtained upon each clinic visit. Cardiac monitor involved 12-lead electrocardiogram, 24-hour Holter recording, and TTE. Additional 12-lead ECG or 24-hour Holter recordings were obtained if patients complained of symptoms of arrhythmia recurrence. Patients included were followed for at least 360 days.

### Definition of endpoints

The primary composite endpoint was defined as: 1. Secondary ablation within 3 months due to the sever early AF recurrence symptoms; 2. Any recurrence of atrial tachycardia (including AF, atrial flutter, and atrial tachycardia) over 30 s during follow-up with or without oral anti-arrhythmic drugs (AADs), AADs for AF were defined as Class I, Class II and Class III antiarrhythmic drugs. Secondary endpoints included all cause death, stroke/TIA, major bleeding events, HF events during follow-up. HF event was defined as an outpatient or inpatient visit to receive treatment for HF. Periprocedural complications, including pericardial effusion, atrial-esophageal fistula, stroke/TIA, major bleeding, and death, were also collected.

### Statistical analysis

All statistical analyses were performed using SPSS v.26.0 statistical analysis software and or R software (version 4.2.2). Categorical variables were described as percentages (%) and tested using the chi-square test, with odds ratio (OR) and 95% confidence interval (CI) also presented. Fisher exact probability method and continuity correction were used for variables not satisfying the chi-square test conditions. Measurement data were tested for normality. Normally distributed data was presented as mean ± standard deviation (X ± s) and tested using the *t*-test. Non-normally distributed data was described using the median (interquartile range) and tested using non-parametric tests (Whitney-Mann tests).

HF and non-HF patients were matched in a 1:1 ratio using propensity score matching (PSM) to reduce treatment-selection bias and potential confounding. We adjusted for age, gender, history of cardiomyopathy, coronary artery disease, hypertension, and EIVOM success which were possibly related to AF recurrence. We calculated a propensity score of 0.1 for maximum execution performance and fixed caliper width. Factors associated with HF were analyzed using logistic regression analyses.

The long-term cumulative survival rates were co using Kaplan-Meier graphs created in GraphPad Prism 9.0 software. Univariate and multivariate Cox regression analyses were used to identify predictors of the recurrence of atrial tachyarrhythmias rate. Univariate and multivariate Cox regression analyses were used to identify predictors of AF recurrence. The numerical variables after the maximally selected Log-rank statistic test were included in the univariate regression analysis. Variables with a *P*-value of less than 0.1 in the univariate analysis and HF status were included in the multivariable model. All tests were two-tailed, and *P*-values less than 0.05 were considered statistically significant.

## Results

### Baseline characteristic

**Figure 1:**
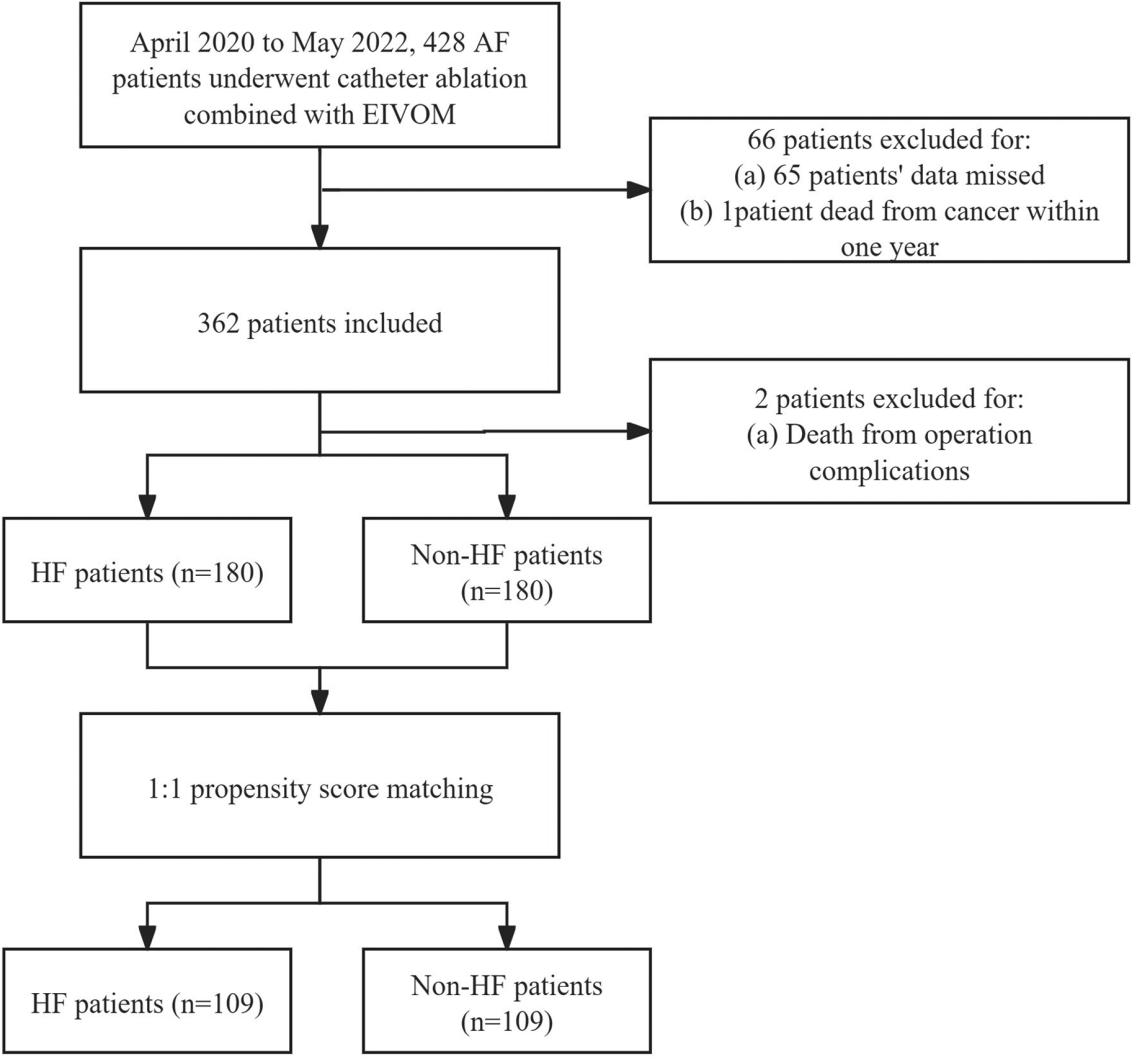
Flow chart of patients who had full record of heart function classification before AF catheter ablation plus EIVOM. AF, Atrial fibrillation; HF, Heart failure; EIVOM, Ethanol infusion of the vein of marshall.

From April 2020 to May 2022, 428 patients received AF ablation combined with EIVOM in a tertiary center, of whom 362 had complete records to assess their heart function before the procedure. 182 had HF and were included in the HF group, 180 were in the non-HF group (Figure 1). Baseline characteristics of the HF and non-HF patients are shown in Table 1.

**Table 1:** Baseline characteristic.

	Before matching			After matching		
	HF (*n* = 182)	Non-HF (*n* = 180)	*P*	HF (*n* = 109)	Non-HF (*n* = 109)	*P*
Age (years)	**62.76** **±** **9.43**	**59.10** **±** **9.33**	**<0**.001**	**62.18** **±** **9.43**	**61.25** **±** **9.06**	**0**.**456**
Male (n,%)	**99** (**54.4%)**	**141** (**78.3%)**	**<0**.001**	**74** (**67.9%)**	**73** (**67.0%)**	**0**.**885**
BMI (kg/m^2^)	25.40 ± 3.53	26.14 ± 10.65	0.378	25.12 ± 3.43	25.54 ± 3.68	0.380
Persistent AF/AFL (n,%)	169 (92.9%)	163 (90.6%)	0.427	94 (93.1%)	89 (87.3%)	0.165
Classification of HF						
HFpEF (n,%)	126 (69.2%)	–		81 (74.3%)	–	
HFrEF (LVEF < 40%) (n,%)	24 (13.2%)	–		12 (11.0%)	–	
HFrEF (LVEF40%–49%) (n,%)	32 (17.6%)	–		16 (14.7%)	–	
Hypertension (n,%)	**106** (**58.2%)**	**76** (**42.2%)**	**0**.002**	**62** (**57%)**	**64** (**58.7%)**	**0**.**784**
Diabetes mellitus (n,%)	25 (13.7%)	18 (10.0%)	0.272	13 (11.9%)	13 (11.9%)	1.000
History of stroke/TIA (n,%)	22 (12.1%)	18 (10.0%)	0.526	11 (10.1%)	13 (11.9%)	0.665
Coronary artery disease (n,%)	44 (24.2%)	36 (20.0%)	0.338	26 (23.9%)	24 (22.0%)	0.747
History of Cardiomyopathy (n,%)	**39** (**21.4%)**	**7** (**3.9%)**	**<0**.001**	**8** (**7.3%)**	**7** (**6.4%)**	**0**.**999**
Dilated cardiomyopathy (n,%)	**25** (**13.7%)**	**0** (**0%)**	**<0**.001**	1 (0.9%)	0 (0%)	1.000
Hypertrophic cardiomyopathy (n,%)	8 (4.4%)	5 (2.8%)	0.408	5 (2.8%)	5 (2.8%)	1.000
Ischemic cardiomyopathy (n,%)	6 (3.3%)	2 (1.1%)	0.284	2 (1.1%)	2 (1.1%)	1.000
Peripheral vascular disease (n,%)	9 (4.9%)	5 (2.8%)	0.285	5 (4.6%)	5 (4.6%)	1.000
NT-ProBNP (pg/ml)	**1,511.10** **±** **1,288.26**	**447.53** **±** **280.87**	**<0**.001**	**1,413.1** **±** **1,337.1**	**472.9** **±** **235.5**	**<0**.001**
NYHA class	**2[2–3]**	**1[1–1]**	<0.001**	**2[2-2]**	**1[1-2]**	**<0**.001**
I (n%)	6 (3.3%)	144 (80.0%)		2 (1.8%)	74 (67.9%)	
II (n%)	130 (71.4%)	36 (20.0%)		84 (77.0%)	35 (32.1%)	
III (n%)	45 (24.6%)	0 (0%)		22 (20.2%)	0 (0%)	
IV (n%)	1 (0.6%)	0 (0%)		1 (0.9%)	0 (0%)	
CHA2DS2-VASc score	**2[3–4]**	**1[0**–**2]**	<0.001**	**3[2**–**4]**	**2[1**–**3]**	**<0**.001**
HAS-BLED score	**1[0–2]**	**1[0–1]**	<0.001**	**1[0**–**1]**	**1[0**–**1]**	0.415
Echocardiography						
LAD (mm)	**45.85** **±** **5.01**	**44.23** **±** **4.97**	**0**.002**	45.22 ± 5.26	44.58 ± 4.67	0.341
RAD (mm)	**43.36** **±** **6.53**	**41.70** **±** **4.97**	**0**.007**	**43.10** **±** **6.49**	**41.62** **±** **5.15**	**0**.033*
LVEDD (mm)	**49.68** **±** **5.94**	**47.73** **±** **3.86**	**<0**.001**	**49.08** **±** **5.00**	**47.59** **±** **3.96**	**0**.008**
LVEF (%)	**49.48** **±** **8.37**	**55.69** **±** **2.89**	**<0**.001**	**50.28** **±** **7.58**	**55.42** **±** **2.88**	**<0**.001**
Medication						
ACEI/ARB/ARNI	24 (13.2%)	19 (10.6%)	0.439	13 (11.9%)	17 (15.6%)	0.432
BETA-Blocker	95 (52.2%)	80 (44.4%)	0.140	60 (55.0%)	54 (49.5%)	0.416
Antiarrhythmic drugs	63 (34.6%)	62 (34.4%)	0.973	35 (32.1%)	41 (37.9%)	0.394
NOAC	171 (93.9%)	175 (97.2%)	0.131	101 (92.7%)	105 (96.3%)	0.235
Spironolactone	**48**(**26.4%)**	**1**(**0.6%)**	**<0**.001**	**23**(**21.1%)**	**0**(**0%)**	**<0**.001**

Statistically significant differences are indicated as *P < 0.05, **P < 0.01.

Bold values indicate statistically significant results deserving special attention.

Values shown are mean ± standard deviation (SD), *n* (%) or median [lower quartile, upper quartile]. *P*-values were calculated by chi-squared test, t test, or Mann-Whitney U test, as appropriate.

Abbreviations: HF, heart failure; OR, odds ratio; CI, confidence interval; BMI, body mass index; AF, atrial fibrillation; HFpEF, heart failure with preserved left ventricular ejection fraction; HFrEF, heart failure with reduced left ventricular ejection fraction; LVEF, left ventricular ejection fraction; TIA, transient ischemic attack; GFR, glomerular filtration rate; NT-ProBNP, N-terminal B-type natriuretic peptide; NYHA, New York Heart Association; LAD, left atrial diameter; RAD, right arial diameter; LVEDD, left ventricular end diastolic diameter; ACEI, angiotensin-converting enzyme inhibitor; ARB, angiotensin receptor blocker; ANRI, angiotensin receptor neprilysin inhibitor; NOAC, novel oral anticoagulants.

Compared with non-HF patients, HF patients were older (62.76 ± 9.43 vs. 59.10 ± 9.33, *P* < 0.001), and were less likely to be male (54.4% vs. 78.3%, *P* < 0.001). They exhibited higher prevalence of hypertension (58.2% vs. 42.2%, *P* = 0.002), thus higher CHA2DS2-VASc score [3[2–4] vs. 1[0–2], *P* < 0.001], and higher HAS-BLED score [1[0–2] vs. 1[0–1], *P* < 0.001].

44 (24.2%) HF patients had histories of cardiomyopathy, including 25 (13.7%) dilated cardiomyopathy patients. 126 (69.2%) were LVEF preserved HF (HFpEF) and 56 (30.8%) were EF reduced HF. The median NYHA heart function class was 2[2–3]. Their average NT-ProBNP level was higher than non-HF patients (1,511.10 ± 1,288.26 vs. 447.53 ± 280.87, *P* < 0.001).

All patients received ultra-sound cardiography on admission. Compared to non-HF patients, HF patients exhibited larger left atrial diameter (LAD) (45.85 ± 5.01 vs. 44.23 ± 4.97, *P* = 0.002), larger right atrial diameter (RAD) (43.36 ± 6.53 vs. 41.70 ± 4.97, *P* = 0.07), greater left ventricular end-diastolic diameter (LVEDD) (49.68 ± 5.94 vs. 47.73 ± 3.86, *P* < 0.001), and lower LVEF (49.48 ± 8.37 vs. 55.69 ± 2.89, *P* < 0.001).

After 1:1 PSM for age, gender, history of cardiomyopathy, coronary artery disease, hypertension, and EIVOM success, 109 cases were included in each group. Baseline characteristic were comparable between the two groups after PSM, while HF-related differences were still statistically significant in NT-ProBNP, NYHA class, CHA2DS2-VASc score, spironolactone medication and echocardiographic measurements (Table 1).

### Procedure and complications

**Figure 2:**
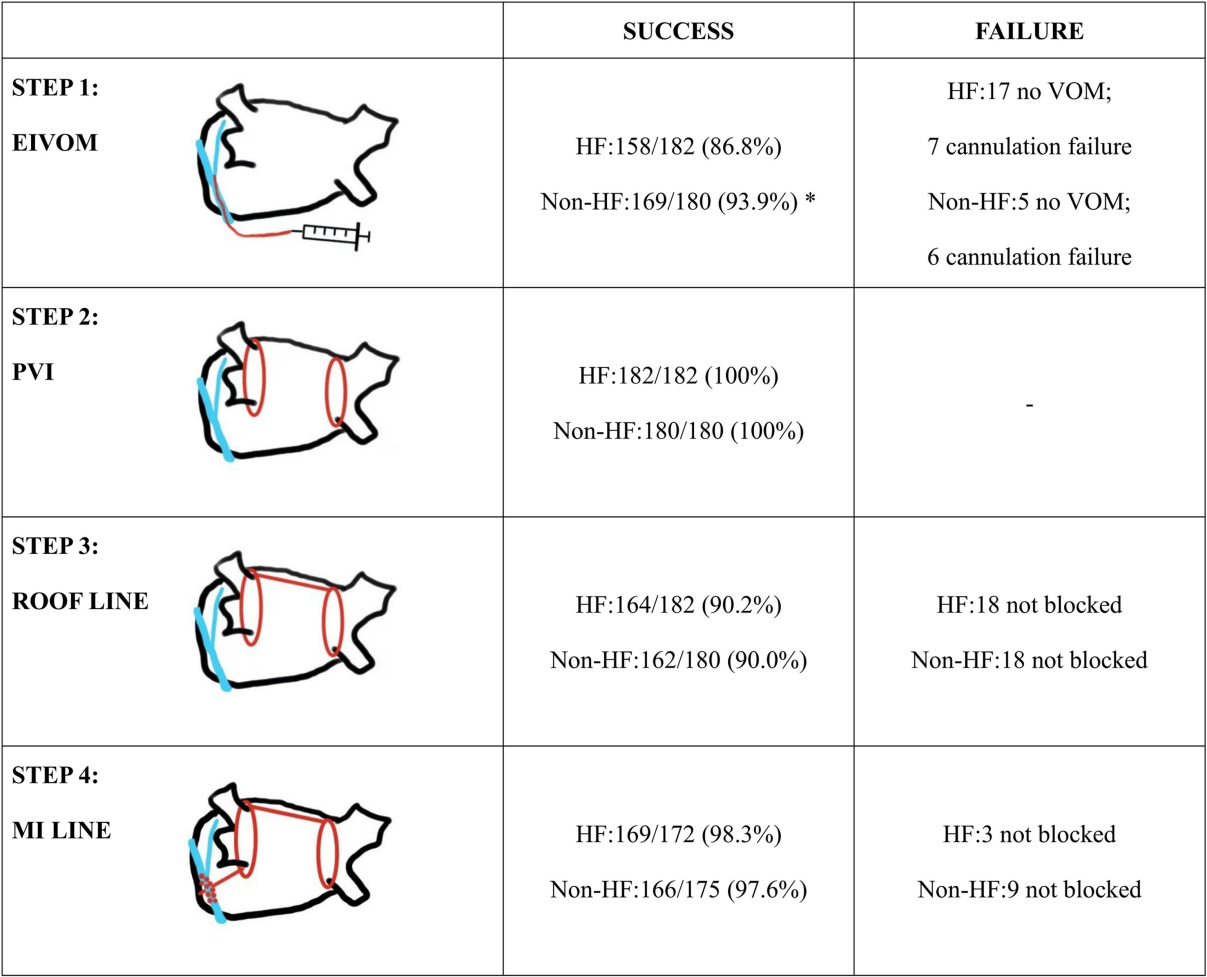
Summary of the ablation strategy and details of success rate in each step. Statistically significant differences are indicated as *P < 0.05, **P < 0.01. Abbreviations: EIVOM, Ethanol infusion of the vein of marshall; VOM, vein of marshall; PVI, pulmonary vein isolation; MI, mitral isthmus.

Table 2 gave the characteristics of the procedure and peri-procedure complications. Of note, HF group showed a significant lower rate of VOM presence upon CS angiography (90.7% vs. 97.2%, *P* = 0.009), and they also showed a significant lower success rate of EIVOM (86.8% vs. 93.9%, *P* = 0.023). All patients achieved PVI success during the procedure. 172 (94.5%) HF patients and 170(94.4%) received MI linear ablation. 111 HF patients and 115 non-HF patients needed additional ablations within the CS to achieve MI block (*P* > 0.05). MI block rates were equally high in both groups (98.3% vs. 97.6%, *P* = 0.691). 164 (90.1%) HF patients and 162 (90.0%) non-HF patients received LA roof linear ablation (*P* = 0.972). AF patients with HF received more linear ablation, attributable to their higher rate of anterior linear ablation (5.5% vs. 0.6%, *P* = 0.011) and tricuspid isthmus linear ablation (13.7% vs. 5.6%, *P* = 0.008). Detailed information about each step is depicted in Figure 2.

**Table 2:** Procedure and complications.

	Before matching			After matching		
	HF (*n* = 182)	Non-HF (*n* = 180)	*P*	HF (*n* = 109)	Non-HF (*n* = 109)	*P*
AF	165 (90.7%)	168 (93.3%)	0.349	101 (92.7%)	102 (93.6%)	0.999
Atrial flutter	17 (9.3%)	12 (6.7%)	0.349	8 (7.3%)	7 (6.4%)	0.999
Redo AF/AFL	16 (8.8%)	17 (9.4%)	0.829	5 (4.6%)	10 (9.2%)	0.181
Presence of VOM	**165** (**90.7%)**	**175** (**97.2%)**	**0**.009**	104 (95.4%)	104 (95.4%)	1.000
EIVOM success	**158** (**86.8%)**	**169** (**93.9%)**	**0**.023*	**101** (**92.7%)**	**98** (**89.9%)**	**0**.**471**
PVI success	182 (100%)	180 (100%)	1.000	109 (100%)	109 (100%)	1.000
MI ablation	172 (94.5%)	170 (94.4%)	0.980	103 (94.5%)	103 (94.5%)	1.000
MI block	169 (98.3%)	166 (97.6%)	0.691	102 (99.0%)	101 (98.1%)	0.561
CS ablation	111 (64.5%)	115 (67.6%)	0.543	66 (64.1%)	69 (67.0%)	0.660
Roof linear ablation	164 (90.1%)	162 (90.0%)	0.972	99 (90.8%)	96 (88.1%)	0.508
Anterior linear ablation	**10** (**5.5%)**	**1** (**0.6%)**	**0**.011*	**5** (**4.6%)**	**0** (**0%)**	**0**.024*
Posterior BOX ablation	13 (7.1%)	8 (4.4%)	0.272	8 (7.3%)	6 (5.5%)	0.581
CAFEs ablation	18 (9.9%)	15 (8.3%)	0.607	8 (7.3%)	11 (10.1)	0.471
Tricuspid isthmus linear ablation	**25** (**13.7%)**	**10** (**5.6%)**	**0**.008**	**13** (**11.9%)**	**5** (**4.6%)**	**0**.049*
Peri-procedural complications						
All Pericardial effusion	80 (44.0%)	67 (37.2%)	0.192	**52** (**47.7%)**	**37** (**33.9%)**	**0**.039*
All pericardial effusion thickness	**8.01** **±** **4.11**	**6.81** **±** **1.93**	**0**.021*	8.35 ± 4.69	6.78 ± 2.20	0.063
Minor pericardial effusion thickness(<10 mm)	6.75 ± 1.54	6.38 ± 129	0.147	6.79 ± 1.53	6.21 ± 1.34	0.090
Major Pericardial effusion(≥10 mm)	13 (7.1%)	6 (3.3%)	0.104	9 (8.3%)	4 (3.7%)	0.153
All Cardiac tamponade	2 (1.1%)	1 (0.5%)	1.000	1 (0.9%)	1 (0.9%)	1.000
Phrenic nerve injury	0 (0%)	1 (0.5%)	0.497	0 (0%)	0 (0%)	1.000
Stroke/TIA	1 (0.6%)	1 (0.5%)	1.000	0 (0%)	0 (0%)	1.000
Atrial-esophageal fistula	2 (1.1%)	0(0%)	0.499	0(0%)	0(0%)	1.000
Death	2(1.1%)	0(0%)	0.499	0(0%)	0(0%)	1.000

Statistically significant differences are indicated as *P < 0.05, **P < 0.01.

Bold values indicate statistically significant results deserving special attention.

Values shown are mean ± standard deviation or *n* (%). *P*-values were calculated by chi-squared test, t test, as appropriate.

Abbreviations: HF, Heart failure; OR, odds ratio; CI, confidence interval; AFL, atrial flutter; VOM, vein of marshall; EIVOM, ethanol infusion of the vein of marshall; PVI, pulmonary vein isolation; MI, mitral isthmus; CS, coronary sinus; CAFEs, complex fractionated atrial electrograms; TIA, transient ischemic attack.

No significant differences in major procedural complications were observed between HF and non-HF patients. Incidences of pericardial effusion as well as cardiac tamponade were numerically higher in HF patients, but the discrepancy did not reach statistical significance. Overall average pericardial effusion depth was deeper in the HF group than that in the non-HF group (8.01 ± 4.11 vs. 6.81 ± 1.93, *P* = 0.021).

There were 2 (1.1%) deaths due to atrial-esophageal fistula in the HF group, which appears to be a high proportion for the size of the study population.

After PSM, the two cases of atrial-esophageal fistula were excluded, while the HF group showed significant higher incidence of pericardial effusion compared to non-HF group. HF patients received more additional linear ablation, such as anterior linear ablation and CTI linear ablation (Table 2).

### Clinical outcomes during follow-up

The events during the follow-up period are described in Table 3. HF patients were followed up for 365 [365–662.5] days, and non-HF patients 365 [365–535.7] days (*P* = 0.058). 44.4% patients remained on AAD medication in the follow-up. This disparity between HF and non-HF patients (53.3% vs. 35.5%, *P* < 0.001) was primarily attributed to differences utilization of beta blockers (46.1% vs. 28.3%). During the follow-up, AF recurrence occurred in 74 (20.5%) patients of all, including 40 (22.2%) HF patients and 34 (18.9%) non-HF patients. Of the recurrences in patients without AAD, 17 (9.4%) were in HF patients and 20 (11.1%) in non-HF patients. Of the total recurrences, 63 (17.5%) recurred after the blanking period of 90 days, there was no statistical significance for any form of recurrences in the HF and non-HF groups.

**Table 3:** Clinical outcomes during follow-up.

	ALL (*n* = 360)	HF (*n* = 180)	Non-HF (*n* = 180)	*P*	OR/HR (95% CI)
Follow-up period (days)	365[365–610]	365[365–662.5]	365[365–535.7]	0.058	**–**
Use of AAD at follow-up	160 (44.4%)	96 (53.3%)	64 (35.5%)	**<0**.001**	2.07 (1.36–3.16)
AAD type					
Amiodarone/Dronedarone	24 (6.7%)	12 (6.7%)	12 (6.7%)	1.000	1.00 (0.50–2.00)
Beta-blockers	134 (37.2%)	83 (46.1%)	51 (28.3%)	0.365	1.22 (0.79–1.89)
Propafenone	2 (0.5%)	1 (0.5%)	1 (0.5%)	1.000	1.00 (0.50–2.00)
Recurrence					
All recurrence	74 (20.5%)	40 (22.2%)	34 (18.9%)	0.683	1.10 (0.70–1.74)
All recurrence without AAD	37 (10.3%)	17 (9.4%)	20 (11.1%)	0.517	1.22 (0.66–2.30)
Recurrence after blanking period	63 (17.5%)	35 (19.5%)	28 (15.6%)	0.561	1.16 (0.71–1.90)
Death					
All-cause death	5 (1.4%)	1 (0.6%)	4 (2.2%)	0.141	0.27 (0.05–1.55)
Death from cardiac causes	1 (0.3%)	0 (0%)	1 (0.6%)	0.257	0.10 (0.00–5.30)
Stroke/TIA	5 (1.4%)	1 (0.6%)	4 (2.2%)	0.136	0.26 (0.05–1.52)
Major bleeding	2 (0.5%)	2 (1.1%)	0 (0%)	0.189	6.46 (0.40–104.7)
HF					
Overall HF	**25** (**6.9%)**	**20** (**11.1%)**	**5** (**2.8%)**	**0**.007**	**3.03** (**1.34–6.88)**
Non-hospitalized HF	18 (5.0%)	14 (7.8%)	4 (2.2%)	0.057	2.60 (0.97–6.91)
Hospitalized HF	7(1.9%)	6(3.3%)	1(0.6%)	0.071	3.91(0.89–17.26)

Statistically significant differences are indicated as *P < 0.05, **P < 0.01.

Bold values indicate statistically significant results deserving special attention.

Values shown are *n* (%) or median [lower quartile, upper quartile].

Abbreviations: HF, heart failure; OR, odds ratio; HR, hazard ratio; CI, confidence interval; AAD, anti-arrhythmic drugs; TIA, transient ischemic attack.

All-cause death occurred in 5 (1.4%) patients, including 1 (0.3%) patient who died from cardiac cause. Stroke or TIA occurred in 5(1.4%) patients, including 1 HF and 4 non-HF patients. Bleeding occurred in 2 (0.5%) patients, which were both gastrointestinal hemorrhages. No significant differences were observed between two groups in term of these events.

Overall HF occurred in 25 (6.9%) patients, including 18 (5.0%) non-hospitalized HF and 7 (1.9%) hospitalized HF. Prevalence of overall HF was significant higher in the HF group [hazard ratio (HR) 3.03, Log-rank *P* = 0.007].

**Figure 3:**
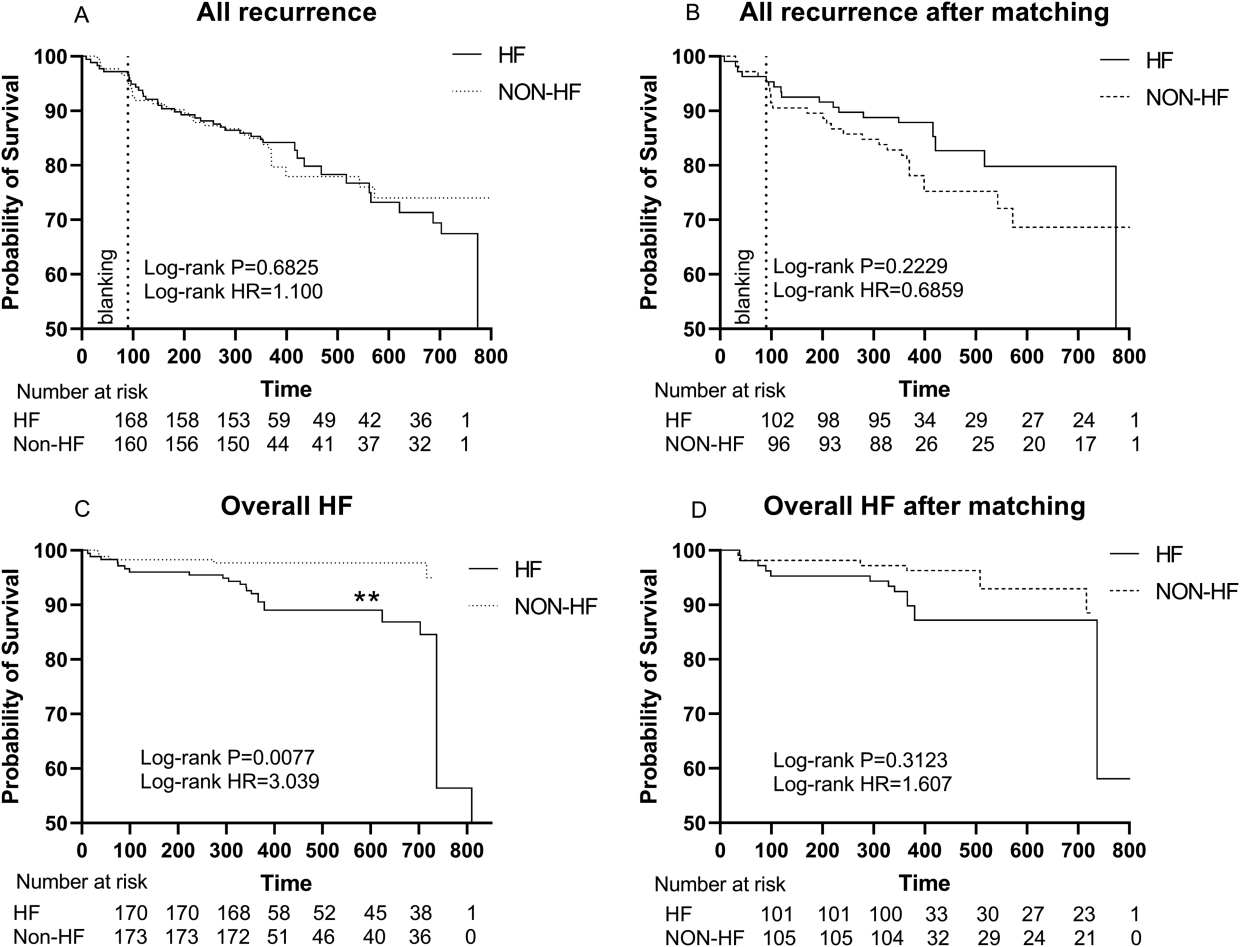
**(A)** Kaplan–Meier graph showing cumulative survival from AF recurrence between patients with and without HF; **(B)** Kaplan–Meier graph showing cumulative survival from AF recurrence between patients with and without HF after matching; **(C)** Kaplan–Meier graph showing cumulative survival from overall HF between patients with and without HF. **(D)** Kaplan–Meier graph showing cumulative survival from overall HF between patients with and without HF after matching. Statistically significant differences are indicated as *P < 0.05, **P < 0.01, ***P < 0.001, ****P < 0.0001. Abbreviations: HF, Heart failure; HR, hazard ratio.

**Figure 4:**
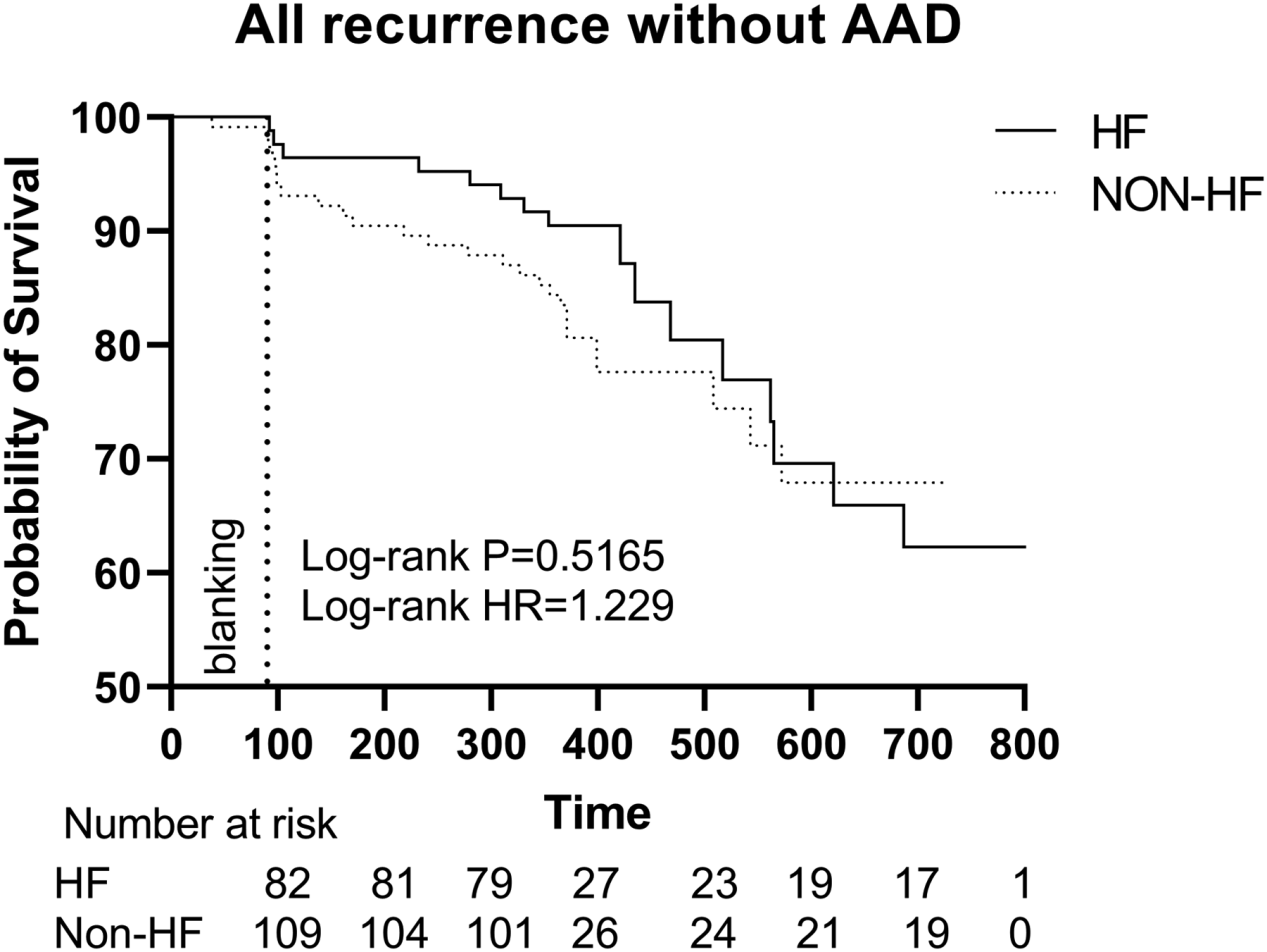
Kaplan–Meier graph showing cumulative survival from AF recurrence between patients with and without HF without AAD**.** Abbreviations: HF, Heart failure; HR, hazard ratio; AAD, anti-arrhythmic drugs.

The Kaplan-Meier survival curve showed no significant difference in the primary endpoints between HF and non-HF group (HR 1.100, Log-rank *P* = 0.6825). Similarly, among patients without AAD, HF group did not show a higher recurrence rate (HR 1.229, Log-rank *P* = 0.5165). However, there were significant differences in overall HF incidence between the two groups (HR 3.039, Log-rank *P* = 0.0077) (Figures 3A,C, Figure 4).

After PSM, Kaplan-Meier survival curve showed no significant difference in the primary endpoints (HR 0.6859, Log-rank *P* = 0.2229) and overall HF (HR 1.607, Log-rank *P* = 0.3123) between HF and non-HF group (Figures 3B,D**).**

### Survival regression analysis

In the univariate Cox regression analysis, female (HR 2.29, 95% CI 1.44–3.63, P < 0.001), age ≥ 59 (HR 1.60, 95% CI 0.99–2.61, *P* = 0.057), GFR ≤ 91 (HR 1.90, 95% CI 1.07–3.36, *P* = 0.028), and LAD ≥ 48 mm (HR 1.69, 95% CI 1.07–2.68, *P* = 0.025) were significant predictors of the primary composite endpoint (Table 4A).

**Table 4:** Cox regression table.

Variables	Univariate		Multivariable	
	HR (95% CI)	*P*	HR (95% CI)	*P*
(A) Cox regression table before matching				
HF	**1.01** (**0.70,1.75)**	**0**.**666**		
Female gender	**2.29** (**1.44,3.63)**	**<0**.001**	**1.52 (1.21,1.93)**	**<0**.001**
Hypertension	0.97 (0.77,1.21)	0.770		
Coronary artery disease	0.81 (0.63,1.05)	0.110		
History of cardiomyopathy	0.81 (0.63,1.04)	0.103		
Age ≥ 59	1.60 (0.99,2.61)	**0**.**057**		
LAD ≥ 48mm	**1.69** (**1.07,2.68)**	**0**.025*	**1.73 (1.08,2.76)**	**0**.022*
EIVOM successful	0.54 (0.20,1.49)	0.234		
Anterior linear ablation	0.86 (0.37,1.98)	0.717		
Tricuspid isthmus linear ablation	1.03 (0.51,2.08)	0.933		
(B) Cox regression table after matching				
HF	**0.70** (**0.38,1.28)**	**0**.**246**		
Female gender	**1.99** (**1.07,3.70)**	**0**.030*	**1.99 (1.07,3.70)**	0.030*
Hypertension	1.34 (0.73,2.46)	0.346		
Coronary artery disease	1.03 (0.71,1.50)	0.869		
History of cardiomyopathy	1.72 (0.86,3.45)	0.127		
Age ≥ 59	1.66 (0.85,3.25)	0.136		
LAD ≥ 48mm	1.61 (0.87,3.00)	0.128		
EIVOM successful	1.72 (0.41,7.13)	0.456		
Anterior linear ablation	0.89 (0.53,1.49)	0.648		
Tricuspid isthmus linear ablation	1.05 (0.39,2.85)	0.917		

Statistically significant differences are indicated as *P < 0.05, **P < 0.01.

Bold values indicate statistically significant results deserving special attention.

Abbreviations: HR, hazard ratio; CI, confidence interval; HF, heart failure; LAD, left atrial diameter; GFR, glomerular filtration rate; EIVOM, ethanol infusion of the vein of marshall;.

Multivariate Cox regression analysis identified female and LAD as two independent risk factors predicting primary endpoint events. Being female was associated with a 1.52 times higher risk of AF recurrence (95% CI 1.21–1.93, *P* < 0.001). Similarly, patients with LAD ≥ 48 mm had a 1.73 times higher risk (95% CI 1.08–2.76, *P* = 0.022) (Table 4A).

After PSM, HF was still not a significant risk factor for AF recurrence in cox univariate and multifactorial regression, and female gender remained an independent risk factor (HR 1.99, 95% CI 1.07–3.70, *P* = 0.030) (Table 4B).

### Subgroup analyses of the clinical endpoint events

Primary endpoint events, as well as echocardiography measurements before and after ablation were compared among different HF subgroups.

**Figure 5:**
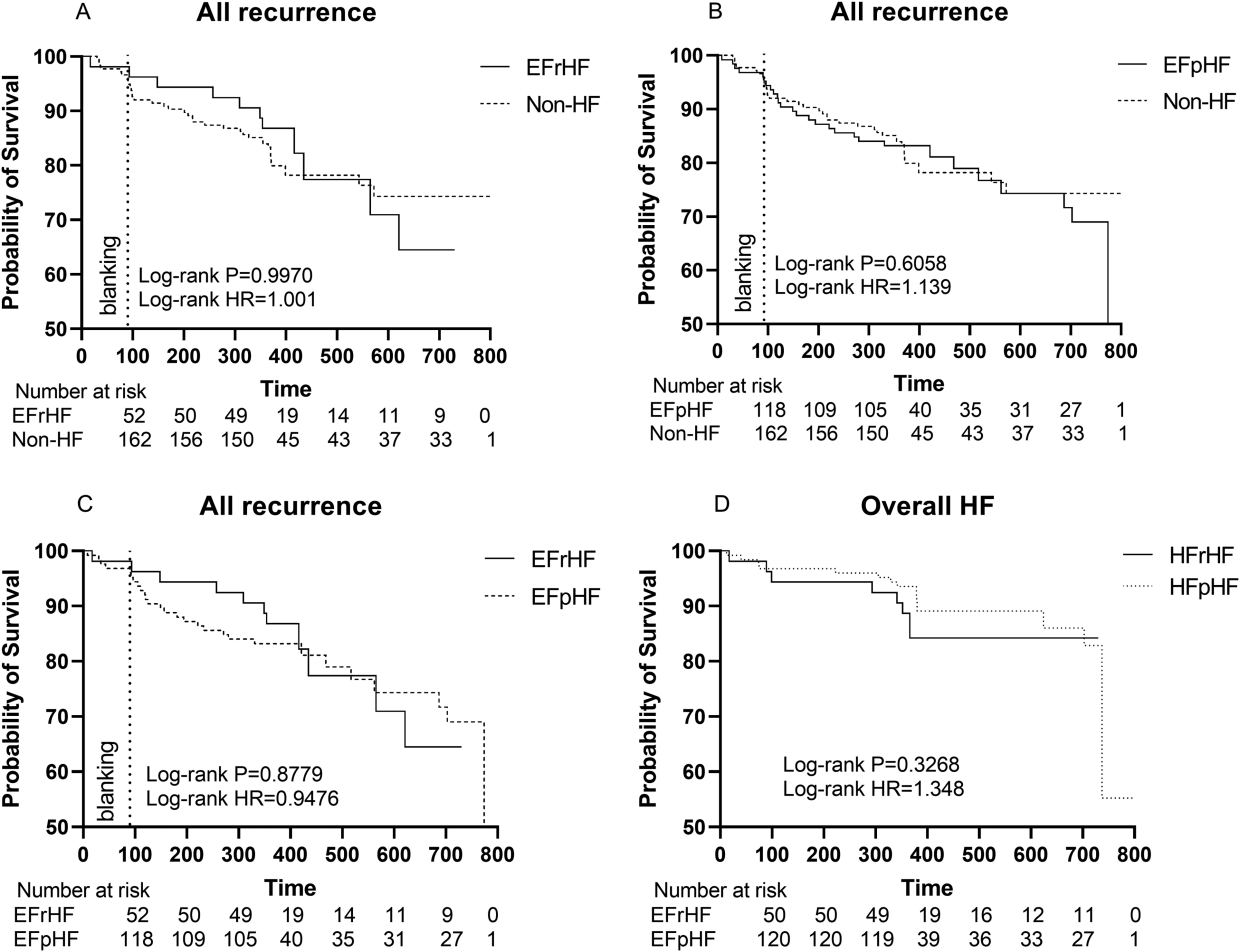
**(A)** Kaplan–Meier graph showing cumulative survival from AF recurrence between HFrEF and non-HF patients; **(B)** Kaplan–Meier graph showing cumulative survival from AF recurrence between HFpEF and non-HF patients; **(C)** Kaplan–Meier graph showing cumulative survival from AF recurrence between HFpEF and HFrEF patients; **(D)** Kaplan–Meier graph showing cumulative survival from overall HF between HFrEF and HFpEF patients. Statistically significant differences are indicated as *P < 0.05, **P < 0.01, ***P < 0.001, ****P < 0.0001. Abbreviation: HF, Heart failure; HR, hazard ratio; HFrEF, Heart failure with reduced left ventricular ejection fractions; including all patients with left ventricular ejection fractions <50%. HFpEF, Heart failure with preserved left ventricular ejection fractions.

The rate of primary endpoints in HFrEF patients was comparable to that of non-HF patients (HR 0.9970, Log-rank *P* = 0.9970, Figure 5A).

Similarly, the rate of composite endpoints in HFpEF patients exhibited no significant difference from that of non-HF patients (HR 1.139, Log-rank *P* = 0.6058, Figure 5B).

Furthermore, there were no significant differences between HFrEF and HFpEF patients (HR 0.9476, Log-rank *P* = 0.8779, Figure 5C).

Also, different HF subgroups (HFrEF or HFpEF) had an equal risk of developing HF during our follow-up period (HR 1.348, Log-rank *P* = 0.3268, Figure 5D).

### Echocardiographic follow-up in HF subgroups

**Figure 6:**
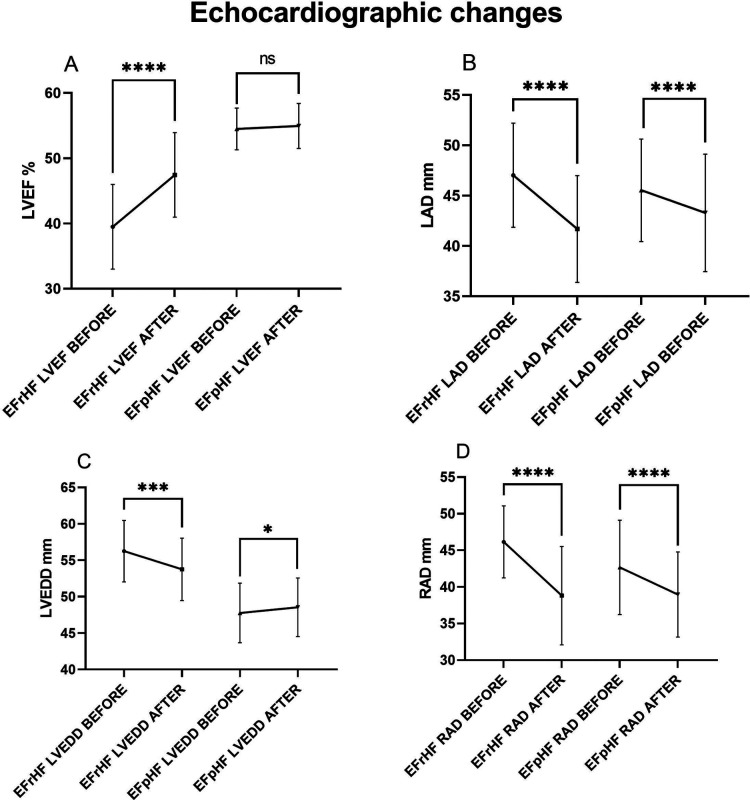
Hfref and HFpEF patients’ echocardiographic comparisons before and after AF ablation plus EIVOM: **(A)** left ventricular ejection fraction, **(B)** left atrial diameter, **(C)** left ventricular diameter, **(D)** right atrial diameter. Values shown are mean ± SD. *P* values were calculated by paired sample *t*-test. Statistically significant differences are indicated as ns, no significance, *P < 0.05, **P < 0.01, ***P < 0.001, ****P < 0.0001. Abbreviations: HFrEF, Heart failure with reduced left ventricular ejection fractions; including all patients with left ventricular ejection fractions <50%; HFpEF, Heart failure with preserved left ventricular ejection fractions; LVEF, Left ventricular ejection fraction; LAD, Left atrial diameter; LVEDD, Left ventricular end diastolic diameter; RAD, Right arial diameter.

Transthoracic echocardiography was repeated 3–6months after ablation in 112(62.2%) of 180 HF patients (Table 5 and Figure 6). Echocardiographic measurement changes showed different patterns in HFrEF and HFpEF patients.

**Table 5:** Echocardiographic changes.

	ALL HF (*n* = 112)		HFrEF (*n* = 32)		HFpEF (*n* = 80)	
	Before	After	Before	After	Before	After
LVEF (%)	**50.21** ± **8.09**	**52.80** **±** 5.63**	**39.50** ± **6.50**	**47.44** **±** 6.48**	54.50 ± 3.20	54.95 ± 3.44
LAD (mm)	**45.96** ± **5.13**	**42.83** **±** 5.71**	**47.03** ± **5.17**	**41.69** **±** 5.31**	**45.54** ± **5.09**	**43.29** **±** 5.83**
LVEDD (mm)	50.19 ± 5.63	50.04 ± 4.71	**56.25** ± **4.22**	**53.75** **±** 4.27**	**47.76** ± **4.10**	**48.55** **±** 4.02*
RAD (mm)	**43.67** ± **6.22**	**38.93** **±** 6.06**	**46.16** ± **4.91**	**38.81** **±** 6.72**	**42.68** ± **6.44**	**38.98** **±** 5.81**

Statistically significant differences are indicated as *P < 0.05, **P < 0.01.

Bold values indicate statistically significant results deserving special attention.

Values shown are mean ± SD. *P*-values were calculated by paired sample *t*-test.

Abbreviations: HFrEF, heart failure with reduced left ventricular ejection fractions; including all patients with left ventricular ejection fractions <50%; HFpEF, heart failure with preserved left ventricular ejection fractions; LVEF, left ventricular ejection fraction; LAD, left atrial diameter; LVEDD, left ventricular end diastolic diameter; RAD, right arial diameter.

Overall, HF patients demonstrated a significant increase in LVEF (50.21 ± 8.09 vs. 52.80 ± 5.63, *P* < 0.001), which was mainly contributed by LVEF improvements in HFrEF patients (39.50 ± 6.50 vs. 47.44 ± 6.48, *P* < 0.001). In HFpEF patients, LVEF showed no significant changes during the follow-up. LVEDD decreased significantly in HFrEF patients (56.25 ± 4.22 vs. 53.75 ± 4.27, *P* < 0.001), while it increased in HFpEF patients (47.76 ± 4.10 vs. 48.55 ± 4.02, *P* = 0.047) suggesting an improved diastolic function. No matter HFrEF or HFpEF, decreases in LAD (45.96 ± 5.13 vs. 42.83 ± 5.71, *P* < 0.001 in all HF), and in RAD (43.67 ± 6.22 vs. 38.93 ± 6.06, *P* < 0.001 in all HF) were consistently observed.

These 112 patients were further divided into recurrence group (*n* = 29) and non-recurrence group (*n* = 83). Overall, patients with no recurrence exhibited better echocardiographic improvements.

Non-recurrence group showed significant improvement in LVEF (50.15 ± 8.17 vs. 52.96 ± 5.71, *P* < 0.001), while LVEF did not improve in the recurrence group (51.03 ± 7.33 vs. 52.79 ± 5.05, *P* = 0.155). Similarly, RAD also showed a significant decrease in the non-recurrence group (43.50 ± 6.13 vs. 37.67 ± 4.94, *P* < 0.001), while the decrease in the recurrence group was not statistically significant (44.07 ± 6.65 vs. 42.38 ± 7.60, *P* = 0.084).

**Figure 7:**
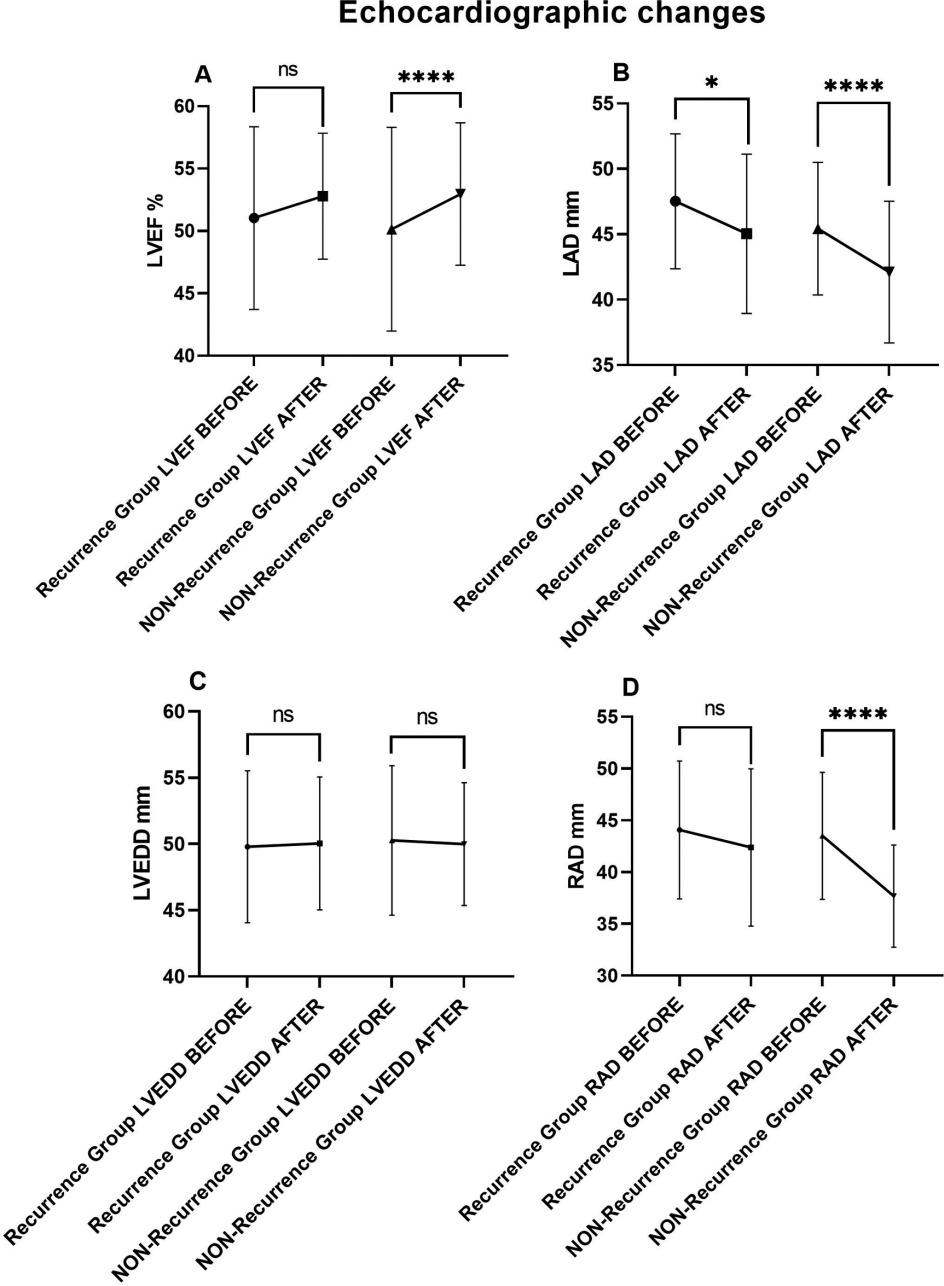
Echocardiographic comparisons in recurrence and non-recurrence HF patients before and after AF ablation plus EIVOM: **(A)** left ventricular ejection fraction, **(B)** left atrial diameter, **(C)** left ventricular diameter, **(D)** right atrial diameter. Values shown are mean ± SD. *P* values were calculated by paired sample *t*-test. Statistically significant differences are indicated as ns, no significance, *P < 0.05, **P < 0.01, ***P < 0.001, ****P < 0.0001. Abbreviations: LVEF, Left ventricular ejection fraction; LAD, Left atrial diameter; LVEDD, Left ventricular end diastolic diameter; RAD, Right arial diameter.

Although both groups showed LAD improvement (47.52 ± 5.17 vs. 45.03 ± 5.07, *P* = 0.014 in recurrence group) (45.43 ± 5.07 vs. 42.11 ± 5.42, *P* < 0.001 in non-recurrence group), the reduction was more substantial in the non-recurrence group (3.317 vs. 2.483). There was no significant change in LVEDD regardless of AF recurrence (49.79 ± 5.73 vs. 50.03 ± 5.02, *P* = 0.756) (50.27 ± 5.64 vs. 49.99 ± 4.64, *P* = 0.497). (Table 6 and Figure 7).

**Table 6:** Echocardiographic changes.

	Recurrence group (*n* = 29)		Non-recurrence group (*n* = 82)	
	Before	After	Before	After
LVEF (%)	51.03 ± 7.33	52.79 ± 5.05	**50.15** ± **8.17**	**52.96** **±** 5.71**
LAD (mm)	**47.52** ± **5.17**	**45.03** **±** 5.07*	**45.43** ± **5.07**	**42.11** **±** 5.42**
LVEDD (mm)	49.79 ± 5.73	50.03 ± 5.02	50.27 ± 5.64	49.99 ± 4.64
RAD (mm)	44.07 ± 6.65	42.38 ± 7.60	**43.50** ± **6.13**	**37.67** **±** 4.94**

Statistically significant differences are indicated as *P < 0.05, **P < 0.01.

Bold values indicate statistically significant results deserving special attention.

Values shown are mean ± SD. *P*-values were calculated by paired sample *t*-test.

Abbreviations: LVEF, left ventricular ejection fraction; LAD, left atrial diameter; LVEDD, left ventricular end diastolic diameter; RAD, right arial diameter.

## Discussion

The main findings of this study are:

(1) For AF patients with HF history, catheter ablation of AF with EIVOM can be completed with comparable success rate to that of non-HF patients.
(2) HF status before ablation increases peri-procedural cardiac effusion rate, and incidence of atrial-esophageal fistula in our study deserves special attention.
(3) Female gender is an independent risk factor for AF recurrence after EIVOM combing catheter ablation.

AF can impair heart pump performance through the loss of LA contraction, inadequate left ventricular diastolic function, as well as mitral or tricuspid valve regurgitations. Sartipy et al (14). reported an association between AF and increased risk of all-cause death, HF hospitalization, and stroke/TIA in patients with HF, in all ejection fraction groups. Studies investigating catheter ablation in AF with concomitant HF patients showed that failure in maintaining sinus rhythm (SR) tended to have a poorer prognosis (15, 16). It is generally accepted that maintaining SR through catheter ablation in AF and HF patients is helpful in improving clinical outcomes (17).

Yet there are concerns over safety events and AF recurrence considering catheter ablation in AF and HF patients. Previous studies yielded mixed results. Maura M Zylla et al (18). found that patients with HFpEF more often experienced AF recurrence, repeat AF ablation, and AF-related re-hospitalization compared to patients without HFpEF. Another study also showed that patients with HF were more likely to experience AF recurrence within both 3 months and 1 year (19). At the same time, there are also studies showing comparable SR maintenance rate among HF and non-HF patients (20, 21).

However, EIVOM was seldom explored in AF patients complicated with HF. VENUS (Vein of Marshall Ethanol for Untreated Persistent AF) trial showed freedom from any clinical AF or atrial tachycardia on monitoring was higher in patients randomized to vein of Marshall–catheter ablation group, compared to catheter ablation alone. The absolute difference reached 11.2% (13).

In our study EIVOM plus catheter ablation (PVI + linear ablation) showed AF burden comparable to that of non-HF patients. The mechanisms of the rhythm control effect by EIVOM may be related to enhanced atrial denervation, or elimination of AF triggers (22, 23). LOM is richly innervated with autonomic nerve fibers and ganglion cells. From the distal to the proximal end of LOM, a decrease in the sympathetic nerve fibers and an increase in the parasympathetic ganglions were observed (24, 25). High-frequency stimulation of the proximal portion of the LOM preferentially induced AF (26). In one study of a canine model, LOM ablation inhibited atrial electrical remodeling during short-term rapid atrial pacing (RAP), as well as eliminated AF induction (27). Study also shows that this anti-arrhythmic effect of LOM ablation was probably due to its parasympathetic denervation effects (28). In clinical practices, LOM is frequently targeted in cardiac ganglion plexi ablations, with positive results of ganglion plexi ablation in controlling AF, with positive effects (29). Given the distinctive feature of Marshall bundle being abundant in sympathetic nerve fibers, an intriguing hypothesis presents itself that EIVOM might exhibit independent advantages in reversing cardiac remodeling in HF patients, apart from its anti-arrhythmic impacts. Currently, however, the clinical evidence remains remarkably sparse. A study conducted by Fabrizio R Assis et al (30). showed that cardiac sympathetic denervation effectively decreased ventricular tachycardia (VT) recurrence in HF patients, which is a major cause of sudden cardiac death. In our follow-up of HF patients, they also exhibited a mortality rate similar to those without HF. Further rigorous randomized investigation might be beneficial to validate this proposition.

EIVOM also facilitates the success rate of MI bidirectional block, which plays a crucial role in reducing recurrence after AF ablation (31). MB mediated re-entrant atrial tachycardia accounted for up to 30.2% of the left AT post AF ablation (32, 33). MI linear ablation is helpful in maintaining SR after AF ablation. However, it's hard to achieve bidirectional mitral isthmus block for the epicardial MB connections spanning this area. EIVOM combined with endocardial and CS ablation greatly improves the success rate of perimetral block, which is in concordance with our results (13, 34).

Despite different echocardiographic improvement patterns in HFrEF and HFpEF patients, there was a significant reduction in LAD and RAD for both groups during the follow-up, indicating an enhanced atrial performance could potentially underlie the benefits derived from AF ablation. AF is frequently associated with pathological atrial myocardial dysfunction and remodeling. LA myocardial dysfunction in AF patients presents as LA dilation and LA fibrosis (35). Since LA is in direct communication with the pulmonary veins, increased LA pressure can result in hydrostatic pulmonary edema (36). Successful SR maintenance plays an important role in reversing the atrial remodeling, as well as in reducing the pulmonary edema. Additionally, it has shown a high rate of freedom from arrhythmia recurrence that is comparable to non-HF patients at the follow-up period.

An atrio-esophageal fistula usually results from heart surgery or other medical procedures, with high death rate. Atrial-esophageal fistula is not a common complication in AF ablation (37). But the incidence of atrial-esophageal fistula in our study deserves special attention. Currently there is no evidence that EIVOM is related with esophageal injury. The 2 cases of atrial-esophageal in our study might be accidental.

The anatomic lateral LA is remote from esophagus. The blood supply of VOM is not connected to the vessels of esophagus. So, the likelihood of EIVOM contributing to esophageal damage is negligible. Our results do not provide relationships between EIVOM and incidence of esophagus injury. The two cases might be more likely related with catheter ablation of LA post wall (LA roof line or LA BOX ablation). Novel ablation techniques are being explored to reduce LA and esophageal injury very high-power short duration radiofrequency ablation (vHPSD) and (pulsed field ablation) PFA, as new technologies, demonstrated higher safety compared to traditional radiofrequency ablation techniques. The study of Paolo Compagnucci compared two ablation techniques: vHPSD and standard power radiofrequency ablation. In patients with persistent AF, vHPSD showed a significant advantage in procedure time, and the two groups performed similarly in terms of major safety outcomes, such as the incidence of major complications, and no major adverse events, including esophageal injury (38). The article of explored the safety of PFA and vHPSD. This study showed that the two techniques had similar rates of major safety outcome events, indicating that both PFA and vHPSD have good safety profiles (39). Another study focused on the application of PFA in left atrial posterior wall ablation. Researchers evaluated the penetration and damage after ablation through direct epicardial mapping, showing that PFA achieved transmural isolation in one patient without severe complications. This indicates that PFA has a lower risk of damaging surrounding structures, such as the esophagus. However, this is a pilot study enrolling only 4 patients. Further studies with larger sample sizes and randomized trials are needed to further verify the safety of PFA (40).In future research, conducting larger-scale randomized controlled trials is essential to further validate the long-term safety and efficacy of these ablation techniques. This will not only provide strong support for clinical practice but also confirm the applicability of these treatment methods in a broader population.

For HF patients, safety should be a concern when EIVOM combining catheter ablation is performed. In the meantime, the rate of pericardial effusion is high compared to previous studies (10, 12, 13). This may be due to diagnostic criteria. We recorded any cardiac effusion visible in pericardial echocardiography. Most of the pericardial effusion was less than 10 mm, and absorbed simultaneously.

However, it is important to note that whether these results are encouraging or worrying, further research, larger-scale clinical trials, and longer-term follow-up are needed to confirm the effectiveness, safety, and durability of this novel ablation strategy.

## Conclusion

EVIOM combining catheter ablation can be completed with comparable success rate in AF patients with or without HF. HF group had higher rate of peri-procedural cardiac effusion, which might be attributed to more linear ablation and higher pulmonary capillary wedge pressure (PWCP) related with HF status. 2 cases of atrial-esophageal fistula were observed in the HF group, which also deserves special attention. Thus peri-procedural safety is a concern for HF patients undergoing EIVOM combing AF catheter ablation. During the follow-up, HF status before ablation is not related with increased AF recurrence.

### Limitation

This study is a retrospective, non-randomized study, and is prone to selective bias. The establishment of HF diagnosis primarily depends on patients' clinical manifestations, echocardiography measurements and NT-proBNP levels. Hemodynamic evaluations, such as LAD pressure and PWCP are not available in this study; consequently, possible underrepresentation of subclinical HF patients may exist.

## Data Availability

The raw data supporting the conclusions of this article will be made available by the authors, without undue reservation.
